# Building the evidence to address disparities in type 1 diabetes (BEAD-T1D): methods and design of a study examining barriers and promoters to diabetes device use in families with public insurance

**DOI:** 10.3389/fendo.2026.1815131

**Published:** 2026-04-27

**Authors:** Lauren E. Figg, Ricardo Medina Peñaranda, Daniel Garfias Silva, Sarah Hanes, Gary M. Shaw, Lisa J. Chamberlain, Diana Naranjo, Jennifer K. Raymond, David M. Maahs, Korey K. Hood, Ananta Addala

**Affiliations:** 1 Department of Pediatrics, Division of Pediatric Endocrinology, Stanford University, Stanford, CA, United States; 2 Department of Pediatrics, Stanford University School of Medicine, Stanford, CA, United States; 3 Department of Pediatrics, Division of Pediatric Endocrinology, Children’s Hospital of Los Angeles, University of Southern California, Los Angeles, Los Angeles, CA, United States; 4 Stanford Diabetes Research Center, Stanford University, Stanford, CA, United States

**Keywords:** automated insulin delivery, clinical trials, continuous glucose monitoring, diabetes technology, health equity, type 1 diabetes, underrepresented populations

## Abstract

Continuous glucose monitoring (CGM) and automated insulin delivery (AID) systems have led to improved outcomes in type 1 diabetes (T1D). Diabetes technology use in minoritized populations is 50% lower than more privileged groups. Tailored, multi-factorial interventions are needed to address disparities and improve technology uptake in minoritized youth with T1D. The Building the Evidence to Address Disparities in Type 1 Diabetes (BEAD-T1D) Study assesses drivers of disparities in CGM and AID use in youth with T1D and public insurance to develop an intervention to increase uptake of diabetes technology. This manuscript describes the rationale, design, and protocols of the study. BEAD-T1D is a prospective, mixed-methods study grounded in the social-ecological model informed by sequential triangulation. Study Aim 1 constructs an evidence base of barriers and promoters to CGM and AID use in youth with T1D and public insurance to formulate and test a pilot intervention to increase device uptake in minoritized populations. Study Aim 2 constructs an evidence base of barriers and promoters to recommending devices to youth with T1D and public insurance to formulate and test a pilot intervention for healthcare providers to increase recommendations of devices. The primary outcome is diabetes technology acceptance analyzed via descriptive statistics and univariate analyses to inform the systematic building of a multivariable model. BEAD-T1D lays the groundwork for future efforts to reduce disparities in the uptake and continued use of diabetes technology in marginalized populations. Interventions effective in increasing the uptake and continued use of diabetes technology in youth with T1D and public insurance are necessary to mitigate disparities.

## 1 Background and aims

Type 1 diabetes (T1D) is a chronic, autoimmune disease impacting nearly 4 in every 1000 youth in the United States. T1D requires daily self-management, including delivery of exogenous insulin, as well as consistent monitoring of blood glucose (1, 2). The American Diabetes Association sets guidelines for youth with T1D and establishes a hemoglobin A1c of <7.0% as target (3). Glycemia aligned with this target range can improve short-term safety, such as fewer instances of severe hypoglycemia and diabetic ketoacidosis, and decrease long-term complications, such as cardiovascular disease and retinopathy (4). Recent advances in diabetes technology used to manage T1D, specifically continuous glucose monitoring (CGM) and automated insulin delivery (AID) systems revolutionized health outcomes in diabetes with improvements in glycemia, complications, and quality of life (5–11).

However, diabetes technology utilization is 50% lower in minoritized populations, including lower socioeconomic status and racial and ethnic minority groups, compared to more privileged groups and this gap has worsened over the last decade (12–18). Minoritized groups have historically been underrepresented in research on novel diabetes devices, as most diabetes device studies have been conducted with samples of >80% non-Hispanic white (NHW). Thus, the current innovations have not included minoritized youth and families’ unique perspectives. The upstream drivers of these disparities have been under explored, as have opportunities to address the drivers of these disparities (19–22). There is a significant need to develop culturally and linguistically tailored interventions to address these disparities, to equitably improve outcomes for all youth with T1D across the sociodemographic spectrum, as well as include experiences of individuals who have historically been underrepresented in research.

The Building the Evidence to Address Disparities in Type 1 Diabetes (BEAD-T1D) Study was developed to first assess upstream drivers of disparities in CGM and AID use in youth with T1D and public insurance. BEAD-T1D aimed to better understand disparities in T1D and to develop a stakeholder-informed intervention to increase uptake of diabetes technology in this population. Informed by pilot study findings, BEAD-T1D hypothesizes that disparities in diabetes technology use are multi-factorial and rise from system-level factors (e.g. insurance barriers, healthcare system navigation barriers, and clinician bias), interpersonal factors (e.g. experiences of discrimination and community attitudes and beliefs), and individual factors (e.g. parent or youth preferences and health literacy) (23–28). These pilot findings laid the foundation for the current, prospective, mixed-methods, two-phase study in youth living with T1D. BEAD-T1D utilizes quantitative and qualitative data, along with evidence from current literature, to identify themes in diabetes technology use and develop an intervention that addresses these themes and is designed to increase uptake in technology. BEAD-T1D aims to improve representation of diverse groups in clinical research, address the gap in literature relating to barriers and promoters of devices use in youth with T1D and public insurance, and importantly, further clarify the connection between the experience of discrimination and technology acceptance. We hypothesize that (1) youth with public insurance will have an interest in using diabetes technology; (2) their barriers to diabetes technology use will be different than those previously described in high SES youth; (3) family-provider relationship, community, and system-level barriers will be greater in publicly insured youth compared to reports in the literature, and; (4) a pilot intervention co-developed by families will improve diabetes technology attitudes and therefore, uptake of technology. We also hypothesize that for youth with public insurance, healthcare providers will (1) cite significant barriers in recommending diabetes technology; (2) identify unique barriers to diabetes technology recommendation; (3) report family-provider relationship (i.e. implicit bias), community, and system-level barriers, and; (4) more likely recommend diabetes technology to youth with T1D and public insurance after receiving a pilot intervention aimed at increasing rates of technology adoption. The purpose of this manuscript is to describe the rationale, design, and protocols of the BEAD-T1D Study.

## 2 Methods

### 2.1 Study development and design

**Figure 1:**
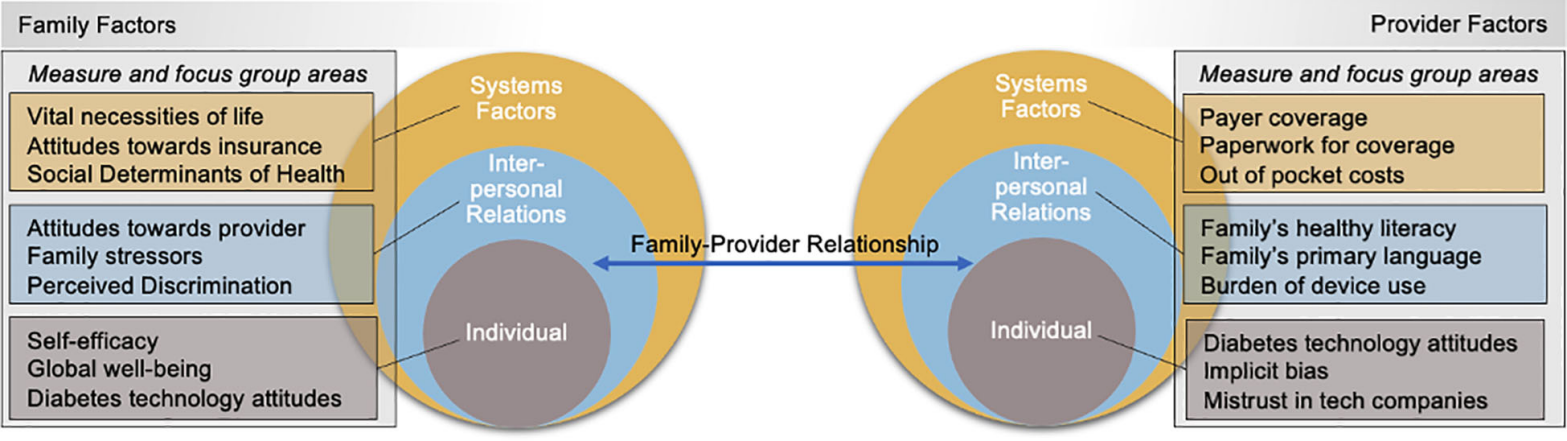
The social ecological model: a theoretical model of human behavior and change.

Building on the study design of BEAD-Pilot, BEAD-T1D uses the social-ecological model (29–32) (**Figure 1**) as a conceptual framework and aimed to address four distinct and critical domains (sociodemographics, experiences of discrimination, mental health and psychosocial factors, and technology acceptance) to understanding drivers of disparities in T1D. The social-ecological model, which is primarily used in public health, presents a holistic view of how different aspects of the environment intersect and influence health behaviors and outcomes. It is inclusive of not only individual factors, but also interpersonal relationships, community, and broader systems factors (30). The primary objective of BEAD-T1D is to assess drivers of disparities in CGM and AID use in youth with T1D and public insurance to understand T1D disparities. The study is a prospective, mixed-methods study informed by sequential triangulation (33) with two aims and two phases. The sequential triangulation is operationalized through quantitative and qualitative data collection (phase 1). Findings will be informed and integrated in a pilot intervention (phase 2) through convergent triangulation.

In Phase I, BEAD-T1D seeks to construct an evidence base of barriers and promoters to CGM and AID use in youth with T1D and public insurance as experienced by families (Aim 1) and clinicians (Aim 2). The primary outcome for both families and clinicians will be technology acceptance, which will be measured using the Diabetes Technology Acceptance (DTA) scale (modified for clinicians). Using a mixed-methods study design theoretically grounded in the social-ecological model (29–32), these two separate populations will inform Phase I through validated surveys, focus groups, and an iterative, stakeholder-informed process. Quantitative and qualitative data are being collected simultaneously, so one does not inform the other and are instead interpreted together. The use of validated surveys adds rigor to the intervention development, and learning through lived experiences adds depth to the process. However, learnings from our pilot study (23–28) informed the qualitative data collection, notably, focus group script development, for the current study.

Findings taken together and analyzed from both methods of data collection will inform the development and pilot testing of a tailored intervention that addresses these themes to increase CGM and AID uptake in minoritized families and healthcare providers to increase recommendations of CGM and AID. Themes for the intervention will be based on themes from data collection (e.g. if technology acceptance is low, intervention content may feature sections that aim to increase that acceptance). Phase II will allow the study team to test acceptability and feasibility of the pilot intervention with both families and healthcare providers.

The study was approved by Stanford University School of Medicine’s Institutional Review Board (#65313) and was registered on ClinicalTrials.gov (NCT05488119). Participants provided informed consent or assent prior to participation and were compensated for their participation. Youth and parent participants each received $50 for survey completion, $100 for focus group completion, and $150 for advisory board completion. Healthcare providers received $50 for survey completion and $100 for focus group completion.

### 2.2 Participants

**Figure 2:**
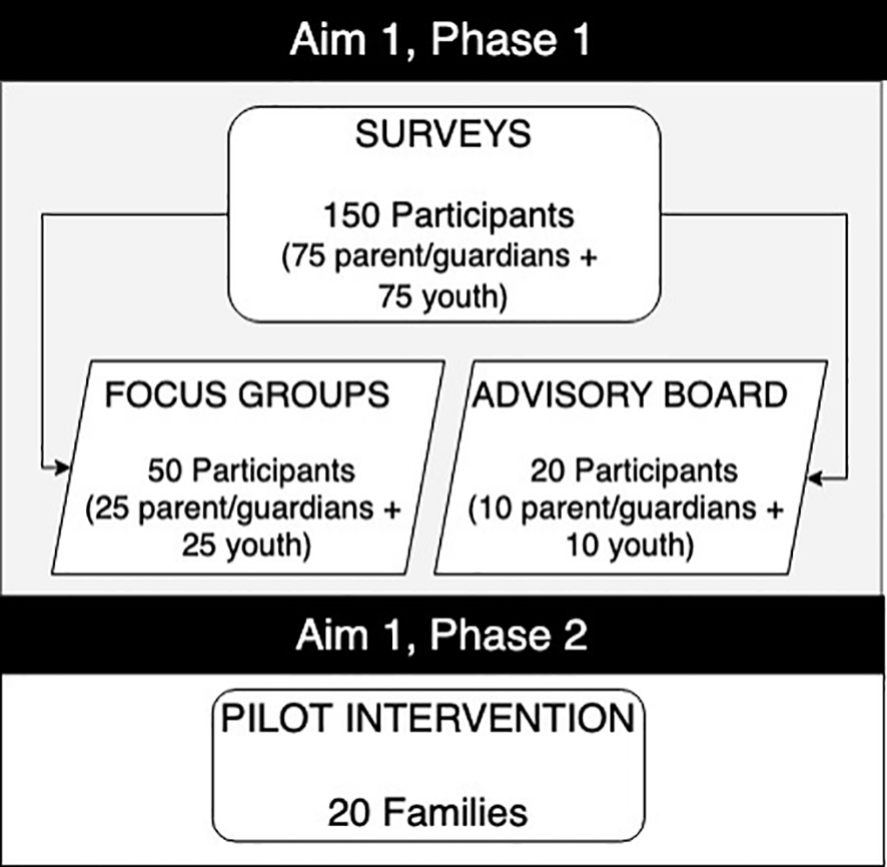
Aim 1 participation goals.

As part of Aim 1 (**Figure 2**), 75 participants (parent/guardian and youth) were recruited as dyads to understand barriers and promoters of technology use at the family level. Based on the disparities in diabetes technology use by SES, sample size calculation to detect a difference in diabetes technology acceptance is 25 participants to achieve a power of 0.8 with an alpha of 0.05. Inclusion criteria for youth included age of 12–21 years, diagnosis of T1D, and utilizing public insurance. Youth also were required to live with the parent/guardian, even if they were over the age of 18, for the purpose of the study and analysis. Inclusion criteria for parent/guardians included caregiving for a youth aged 12–21 years with T1D utilizing public insurance. Parent/guardians were required to provide care for and live with youth, even if they were over the age of 18, for the purpose of the study and analysis. Informed by the pilot data (27) and to meet enrollment goals after facing recruitment challenges in reaching the target population, the study team decided to broaden the inclusion criteria mid-way through the study to create a comparator group to include any type of insurance, including private. Families from marginalized racial and ethnic backgrounds were prioritized as it aligned with the aims.

**Figure 3:**
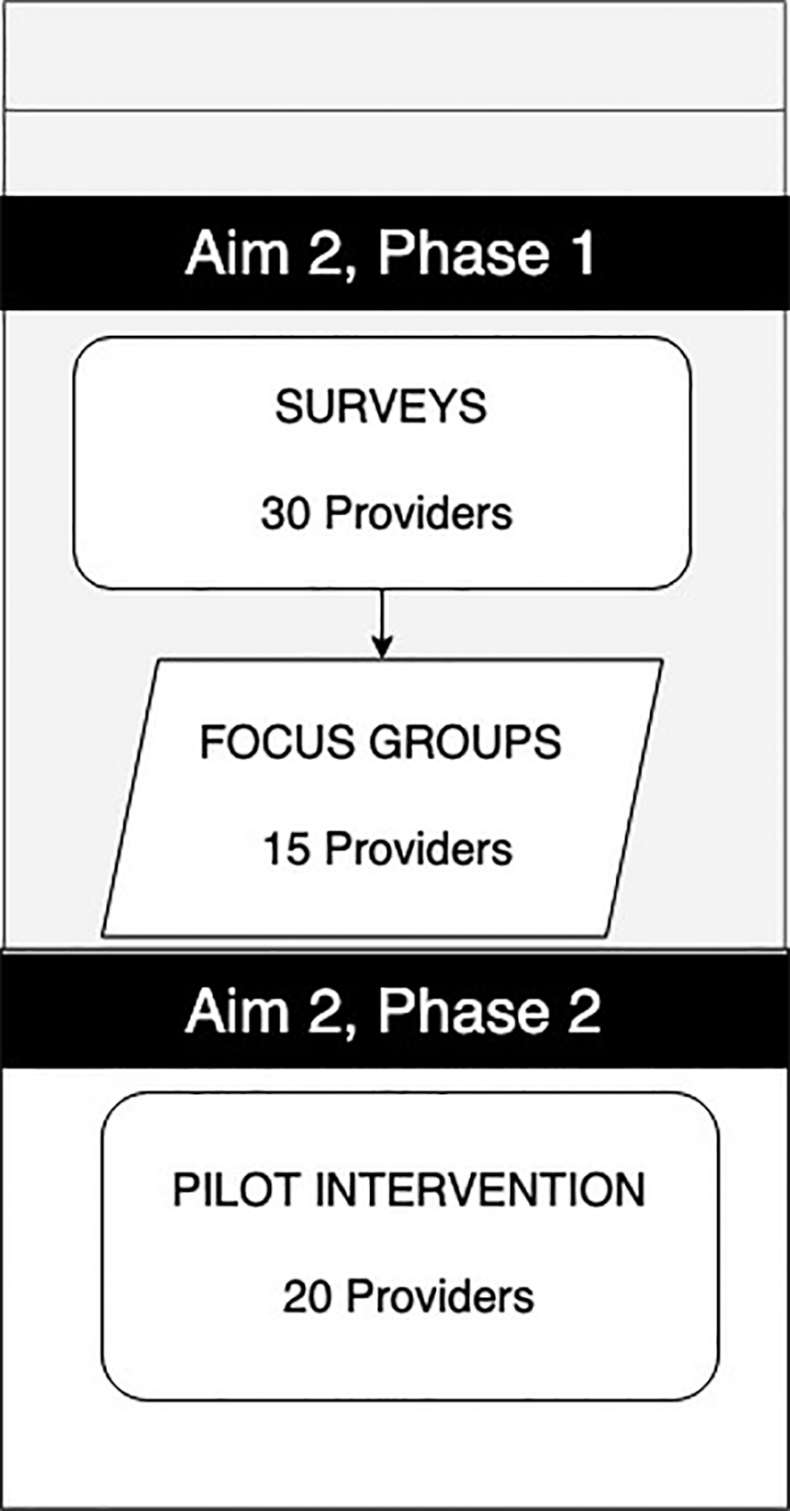
Aim 2 participation goals.

The study aimed to recruit 30 pediatric diabetes healthcare providers as part of Aim 2 (**Figure 3**). Inclusion criteria included clinicians (Physicians, Advanced Practice Providers, or Certified Diabetes Care and Education Specialists) who provide care to youth with T1D between the ages of 12–21 enrolled in public insurance. The specific certifications were chosen because these medical professionals are most likely to recommend and support diabetes technology to families.

### 2.3 Recruitment

Recruitment for Aim 1 was initiated at Stanford Medicine Children’s Health outpatient pediatric endocrinology clinics. Individuals who met inclusion criteria were connected to the study team by the treating physician and approached in-person or via telehealth in their preferred language (English or Spanish). Recruitment also occurred nationally throughout the US in pediatric endocrinologists’ clinics and with community partners in the diabetes space. Recruitment between local and national participants varied based on ease of access to the population, trust in Stanford University as a research institution, and familiarity with clinical research as a whole. The process for local recruitment is clearly defined and well-executed in clinical settings. Additionally, because Stanford University is a research institution, it is not uncommon for patients to be introduced to clinical research during regular clinic visits. National recruitment presented challenges without a warm hand-off from a trusted healthcare provider or previous connection to the research team. With no direct access to the target population, we relied on organic interest, like seeing the study flyer posted or word-of-mouth outreach for national participants. An electronic screener, accessed by a QR code on the study flyer, was used to confirm eligibility, then a phone screener was conducted for final eligibility verification. Participants completed an informed consent and assent process with a waiver of documentation and were then enrolled in the study using a REDCap database. The study intentionally positioned coordinators who shared participants’ cultural backgrounds (Hispanic/Latino) and spoke their preferred language (Spanish) as study leads to improve recruitment and retention (23, 26). Utilizing culturally and linguistically congruent staff, flexible recruitment practices, and prioritizing participant availability were paramount to the successful recruitment efforts of this study, and improved recruitment of historically excluded populations in research.

Recruitment for Aim 2 occurred nationally at academic pediatric endocrine centers and through the T1D Exchange. A QR code accessed via the study flyer allowed eligible participants to complete a waiver of documentation informed consent form and enroll in the study.

### 2.4 Phase I: mixed-methods study design

#### 2.4.1 Quantitative data

For families, surveys were delivered electronically via REDCap to participants at baseline. Surveys explored the four main domains of the study: 1) sociodemographics; 2) experiences of discrimination; 3) mental health and psychosocial factors; and 4) technology acceptance (**Table 1**) (34–47). Surveys were delivered in English or Spanish and took 15–20 minutes to complete. The target completion for Aim 1 participant surveys was 75 dyads (n=150) and the actual completion was 80 dyads (n=160). Youth surveys did not differ between adolescents and young adults. Validated surveys that encompassed the entire youth population (ages 12-21) were selected, and youth who lived with parents, even if over the age of 18, were recruited for participation.

**Table 1:** Survey measures to be completed by youth (Y) or parents/guardians (P/G).

Domain	Survey measure	Item #	Y or P/G	Measure description
Social Determinants of Health (SDOH)	Compilation of Social Determinants of Health Surveys	60	P/G	Surveys to assess social determinants of health including necessities of life, health literacy, and medical system perceptions including mistrust
		16	Y	
Experience of Discrimination	Perceived Ethnic Discrimination Questionnaire Community Version- Brief	17	P/G	Evaluates discrimination related stress in domains: exclusion, stigmatization, discrimination at work/school, and aggression
	Everyday Discrimination Scale	9	P/G	Captures the day-to-day and cumulative experience of discrimination with yes/no responses in 6–9 domains
	Lifetime Discrimination Scale	6	P/G	
	Adverse Childhood Events Questionnaire	10	P/G	Assess childhood trauma which has been shown to have long lasting and broad impacts into adulthood
	Perceptions of Racism in Children and Youth	12	Y	Perceptions of racism and discrimination validated in diverse youth ages 8-18
Technology Readiness	Diabetes Technology Acceptance Scale (DTA)	6	P/G & Y	Evaluation of acceptance of diabetes specific technology including insulin pumps and CGM
	Technology Acceptance Scale	6	P/G	Evaluation of acceptance of technology in general
Psychosocial Factors	Patient Health Questionnaire (PHQ)	9	P/G & Y	Evaluation of depressive symptoms
	Diabetes Distress Scale	20	P/G	Evaluation of distress specific to diabetes and diabetes management
	Problem Areas in Diabetes	20	Y	
	PROMIS Global Health	7	P/G & Y	Evaluation of global well-being

Youth measures are validated in the target age group. PHQ is validated in ≥13; if data for age=12 is unreliable, they will be excluded.

For Aim 2, a survey was delivered electronically via REDCap to provider participants at baseline. Provider survey domains included: 1) psychosocial and wellness factors; 2) technology attitudes; 3) patient-provider relationship; and 4) implicit bias (**Table 2**) (48–52). Surveys were delivered in English, took 15–20 minutes to complete. The target completion for Aim 2 participant surveys was 30 clinicians and the actual completion was n=32.

**Table 2:** Survey measures to be completed by providers.

Domain	Survey measure	Item #	Measure description
Work Life	Maslach Burnout Inventory	22	Assess provider burnout in three domains: emotional, depersonalization, sense of personal accomplishment
	Provider and Staff Satisfaction Survey	6	Evaluates workplace satisfaction
Patient-Provider Relationship	Patient - Practitioner Orientation Scale	18	Assess provider beliefs on patient/family engagement in medical decisions
	Health Care Climate Survey- Modified	6	Providers’ perceptions on patient/family autonomy
Technology Attitudes	Diabetes Technology Acceptance Scale-Modified (DTA-M)	6	Evaluation of acceptance of diabetes specific technology including insulin pumps and CGM
	CGM Benefits and Burden Scale- Modified	16	Evaluation of barriers and burden associated with CGM use
	Diabetes Provider Implicit Bias Tool	7	Evaluates provider implicit bias against public insurance to recommend diabetes technology
	PROMIS Global Health	7	Evaluation of global well-being

#### 2.4.2 Qualitative data

For Aim 1, study staff conducted semi-structured focus groups or interviews with both parents and youth participants who consented to the study and indicated interest in this activity at the time of survey recruitment. Scripts for these semi-structured interviews were developed by study staff and informed by evidence from the pilot study, as well as the social ecological model. The four primary domains (sociodemographics, discrimination, technology acceptance, and mental health) were explored with study participants. These sessions were conducted on Zoom in the participant’s preferred language, English or Spanish, by bilingual study staff. Sessions lasted between 60 and 90 minutes, on average. The target enrollment for focus groups was n=50 (25 parents, 25 youth), and participation did not require parent-youth dyads. The actual enrollment for focus groups was n=47 (24 parents, 23 youth). Youth focus groups did not differ between adolescents and young adults. The same script was used for youth regardless of age. Youth living with their parent/guardian, even if over the age of 18, were recruited for participation; this was considered in focus group script development and diabetes management responsibilities were explored. Additionally, semi-structured interviews in the form of parental advisory boards were conducted for participants who indicated interest. The advisory board sessions differed from the focus groups/interviews in that their objective was to elicit stakeholder input for the development of the intervention. This allowed assurance that stakeholder voices were integrated into the construction of the pilot intervention. Scripts for the advisory boards were developed by study staff and supported by a draft of the pilot intervention materials. Participants provided feedback, with strategic and guided questions, on the draft materials and shared opportunities for improvement. Advisory boards were conducted with parents on Zoom in the participant’s preferred language, English or Spanish. Sessions lasted between 60 and 90 minutes, on average. The target enrollment for advisory board sessions was n=20 (10 parents, 10 youth), and the actual enrollment was n=10 (8 parents, 2 youth).

For Aim 2, study staff conducted semi-structured interviews in the form of focus groups or interviews with healthcare providers who consented to the survey and indicated interest in this study activity. Scripts were developed by study staff with similar themes as the parent and youth focus groups, but with a lens geared toward healthcare providers and their practices. These sessions were also conducted virtually and lasted 60–90 minutes, on average. The target enrollment for Aim 2 focus groups was n=15, and the actual enrollment was n=16.

### 2.5 Phase II: intervention development

A family-level pilot intervention is in development, which utilizes a prospective study design and is intended to test feasibility and acceptability. Utilizing mixed-methods data from Phase I, the study team is currently designing a brief, targeted intervention for families of youth with T1D and public insurance using iterative development as a core tenant of the design. The intervention aims to provide a framework for youth and families to address barriers to CGM and AID uptake. Topics will be centered around themes identified from survey and focus group findings (such as CGM use tips), will be grounded in the shared decision-making model (53, 54), and are intended to be delivered in six weekly, virtual sessions. There will also be an intentional community-building component to the program to allow sharing of lived experiences and connection among participants. Feedback from the advisory board sessions on the intervention design and topics will be integrated to ensure acceptability. We aim to recruit 20 families. The intervention is intended to be delivered at the family level, so parents and youth will be invited to participate. The youth inclusion criteria for the intervention will include an age requirement of 12–21 years, diagnosis of T1D, and utilization of public insurance. Inclusion criteria for parent/guardians will include caregiving for a youth aged 12–21 years with T1D with public insurance. Pre- and post-surveys will be administered to understand outcomes of the pilot intervention.

For clinicians, the intervention will utilize a prospective pilot study design and will aim to test feasibility and acceptability informed by mixed-methods data from Phase I. The purpose of this intervention is to provide a framework that supports clinicians in addressing barriers to CGM and AID recommendations. While topics will be informed by survey and focus group findings, no advisory board for clinicians will occur. However, input from the research team including physicians, psychologists, and other professionals will be incorporated to ensure quality. The sessions will also be delivered virtually and will be grounded in the shared decision-making model (53, 54). Pre- and post-surveys will be administered, as well, for this intervention. Recruitment for the Aim 2 intervention will occur nationally, given the accessibility of the virtual content, and will aim to recruit 20 healthcare providers. Intervention inclusion criteria will include clinicians (Physicians, Advanced Practice Providers, or Certified Diabetes Care and Education Specialists) who provide care to youth with type 1 diabetes between the ages of 12–21 who are enrolled in public insurance. Both interventions are still in development, therefore, content and recruitment plans are not yet finalized.

### 2.6 Data analysis plan

The primary outcome of this study is to identify salient barriers and promoters to diabetes technology use in youth with public insurance (as a proxy for low SES) and their parents/guardians. Diabetes technology use, evaluated separately for CGM and insulin pump, will be operationalized via survey responses as “current use” (>50% device use), “intermittent use” (<50% device use), “discontinued” (stopped a device in the last two years), and “never” (never used a device). In the first phase of analysis, we will evaluate the difference in the survey domains by diabetes technology use via a regression model. Identifying differences in those who have ever adopted technology versus those who have not will offer key areas of focus for intervention design. In the second phase of analysis, we will use model selection, such as elastic net regression, to evaluate which survey domains, youth characteristics, and family characteristics are most associated with the DTA Scale. This analysis will offer another vantage point to target the intervention development to promote acceptance of diabetes technology use, a key factor in diabetes technology uptake. As youth and parent/guardian priorities in diabetes tend to differ, multivariate analysis of variance (MANOVA) will be used to determine differences between parents and youth. This analysis will allow for the targeted development of separate youth and parent interventions, if indicated.

The qualitative data will be analyzed in an inductive fashion without *a priori* constructs to limit bias introduced by the researchers given that these associations and questions have not previously been evaluated. The goal of the qualitative analysis is to identify which factors are considered most important in diabetes technology uptake and use for youth with T1D and public insurance as well as their families. Domains and factors that reach thematic saturation in the inductive qualitative analysis will be prioritized in the intervention development. Particular emphasis will be placed on domains identified in both the qualitative and quantitative data.

## 3 Discussion

BEAD-T1D was developed as a, to our knowledge, novel response to the worsening disparities in T1D outcomes and widening gap in the use of diabetes technology in minoritized youth (16). Given the state of diabetes disparities in the United States and the under exploration of unique experiences of minoritized youth, their families, and their clinical care providers (2, 12–28, 52, 55–67), the study team utilized the social-ecological model as a conceptual framework for the design of the study. The social-ecological model considers that individual behavior, including health behaviors, are shaped by and nested within numerous intrapersonal, interpersonal, organizational, community, and policy factors (29–32). Because of the holistic consideration of how these factors influence overall behavior, it is suited to help us understand the drivers of disparities in diabetes device use. We have the opportunity to consider how anything from state-by-state Medicaid policy to how technology is introduced by a provider impacts a family’s readiness to begin and their acceptance of diabetes technology. Further, the theoretical model can help inform a culturally and linguistically aligned intervention to address the drivers of disparities.

There have been few studies that evaluate drivers of disparities in pediatric T1D, and none have considered perspectives of youth, families and clinicians. There continues to be a need for further research that aims to understand and address these drivers. This work is important not only because of the gaps in literature, but also and most importantly, because it allows for integration of stakeholder feedback to inform interventions that drive change. Our study utilized preliminary data from the BEAD-Pilot study (23–28), which assessed barriers and promoters to diabetes technology use in youth less than 12 years of age at Stanford Medicine Children’s Health. However, the current study included a broader population from across the United States in an effort to understand experiences of minoritized families from various communities and with diverse healthcare experiences.

Building on this need, our study’s design utilized mixed-methods data collection. Our innovative data collection methods, which have been informed by the pilot study (23–28), forge a new path to evaluate previously unexplored factors that impact technology use, such as concepts of discrimination and intersectional identities. A mixed-method approach allows a deeper and more nuanced look into unique experiences, coupling validated survey results with sharing of lived experience through interviews. Through our quantitative data collection, we explore four domains: 1) sociodemographics; 2) experiences of discrimination; 3) mental health and psychosocial factors; and 4) technology acceptance. Sociodemographics have allowed us to conduct sub-analyses that can potentially evaluate the impact of intersectionality and learn more about how different minoritized groups start and use diabetes technology. To our knowledge, the experience of discrimination is a novel aspect of this work, and we hypothesize that perceived discrimination is a barrier to technology use. Our study was designed to explore this further. Psychosocial factors have been salient inclusions in our analysis because of the well-documented impact of diabetes on mental health. Finally, technology acceptance has been critical for this work to understand how families perceive and prepare for diabetes technology use. Our qualitative data collection has allowed us to explore unique parent, youth and provider experiences to provide richer insight into barriers and promoters of diabetes technology by way of the same four domains. Many of the validated measures we have collected have not been evaluated in this population and have allowed us to explore complex and nuanced themes that appear across social determinants of health (18, 24, 68).

Our study asked robust sociodemographic characteristics to capture the heterogeneity of our cohort and evaluate the impact of intersectional identities. First, we queried race and ethnicity in a way that is different from the standard presentation from the United States Office of Management and Budget (69). Rather than using a single, combined question to assess both race and ethnicity, we allowed multiple selections for race and ethnicity, as well as the ability to further specify origin within each selection. For example, choosing Hispanic for race and ethnicity would allow further origin selections of Argentinean, Brazilian, Colombian, Cuban, Dominican, Guatemalan, Honduran, Salvadorean, Mexican, Peruvian, Puerto Rican, or Other Hispanic. This allows us to begin evaluating race as a social construct consistent with the experiences unique to each cultural group, rather than lumping heterogenous people together for the purposes of data collection. We also queried immigration generation to explore similarities and differences in technology use across populations and understand how the unique experience of immigration impacts technology uptake. Additional characteristics we captured for this study include parent/guardian education, household income, and language spoken at home as well as immigration generation, allowing for the evaluation of intersectional identities. The depth of these sociodemographic questions is novel and, to our knowledge, the first time it’s being presented in research.

In addition to our detailed sociodemographic measures, BEAD-T1D included perceived discrimination measures in the data collection (37), which is both an important and unique addition. There is significant data to show that discrimination can cause negative health outcomes, but the role of discrimination in the uptake of diabetes devices is under explored. To our knowledge, BEAD-T1D is the one of the first studies to explore the impact of discrimination on the uptake of diabetes technology in youth with T1D. The inclusion of discrimination measures in our data collection has laid an important foundation for understanding how these experiences shape device use. The study’s greatest value is that it builds an evidence base to understand how discrimination impacts device uptake can later inform interventions to address this (at both the family and provider level), then even later change the trajectory of diabetes disparities in the United States.

A central component of our study design has been the inclusion of a culturally and linguistically congruent staff member to facilitate recruitment, enrollment, and all study activities. Data shows that the inclusion of congruent staff contributes to increased participant engagement and overall study success (23, 26). Additionally, all study materials were translated to Spanish and approved by the IRB. We made these decisions to increase trust and rapport with marginalized communities, so we could more effectively learn about their unique experiences with diabetes technology uptake and continued use and consider ways to improve both aspects.

Alongside its strengths, the study has also presented several limitations and challenges. First, study staff, materials, and activities were only available in English and Spanish. While this allowed us to effectively engage with the Spanish speaking population, we had to exclude other languages from participation. This presents a limitation with study findings by reducing generalizability or limiting perspectives based on language spoken. Future expansion of supported languages will provide further insight into experiences of diabetes technology use in diverse populations. Study materials (e.g. recruitment flyers) and validated measures were translated professionally and then back translated, however, the surveys were not formally validated in Spanish. Because the surveys were translated, they do not carry the same validity as the original measures. However, we conducted back-translation retrospective probing to ensure qualitative validation. Although our protocols have effectively incorporated findings from our pilot study (23–26) around recruitment practices and have improved our recruitment overall, we still faced challenges in recruiting minoritized families for study participation. This contributed to our decision to expand the study to include private insurance. Logistical challenges like changing contact information, shared (e.g. among family members) phone number or email addresses, failed email contact due to full inboxes, and limited participant interaction with electronic health record messaging platforms contributed to barriers to recruitment and engagement. Future efforts will continue to plan for and expect longer recruitment periods and higher than typical ongoing outreach to engage enrolled study participants. Although we recruited nationally, most participants were patients at Stanford Medicine Children’s Health. This may impact generalizability, particularly related to Medicaid differences across states and how diabetes technology is covered in different plans. We utilized social media in recruitment but did not effectively reach eligible participants from across the country. We found that partnering with diabetes organizations improved national recruitment efforts, which will inform future recruitment efforts for this group. While youth surveys and focus group questions were chosen to address all ages of youth in our study, we do not plan to analyze specific age-related differences among diabetes management practices or experiences in adolescents versus young adults. There may be unique differences in the age group we have chosen, and this limitation could inform future directions when developing interventions or future studies. A limitation of the study is that it will not evaluate glycemia and any connections between clinical outcomes and experiences with technology. This limits the ability of the study to evaluate the impact of the intervention on real-time quality and safety. Future studies could explore how varied experiences with diabetes technology, experiences of discrimination, and interventions designed to increase technology use in minoritized populations impact long-term outcomes and glycemia. We hypothesize that Phase II of the study could improve health behaviors and technology acceptance and will explore this after it is fully implemented. Further limitations and challenges will be thoroughly evaluated after data analysis and in future publications.

By combining these important aspects of study design, BEAD-T1D has presented a unique opportunity to reach underrepresented populations to explore the drivers of disparities in diabetes technology uptake and utilize a stakeholder-informed model to develop an intervention to improve these disparities. Future directions include a broader evaluation of device initiation rates, glycemic changes, changes in provider recommendation rates, and inclusion of a broader United States population.

## 4 Conclusion

BEAD-T1D is a timely and important study that lays the groundwork for future efforts to reduce disparities in the uptake and continued use of diabetes technology in marginalized populations. With findings from a pilot study (27), BEAD-T1D was adapted to understand the barriers to technology use, and notably, how experiences of discrimination impact technology use. Based on findings from our initial study phase, we will pilot an intervention for feasibility and acceptability for both parents and youth with T1D and public insurance and diabetes care providers. After evaluating feasibility and acceptability in the current study, a full study will later evaluate the intervention outcomes. Interventions effective in increasing the uptake and continued use of diabetes technology in youth with T1D and public insurance are necessary to inform recommendations for successful use in clinical settings.

## Data Availability

The original contributions presented in the study are included in the article/supplementary material. Further inquiries can be directed to the corresponding author.
